# Temporal patterns in road crossing behaviour in roe deer (*Capreolus capreolus*) at sites with wildlife warning reflectors

**DOI:** 10.1371/journal.pone.0184761

**Published:** 2017-09-27

**Authors:** Jim-Lino Kämmerle, Falko Brieger, Max Kröschel, Robert Hagen, Ilse Storch, Rudi Suchant

**Affiliations:** 1 Chair of Wildlife Ecology and Wildlife Management, University of Freiburg, Freiburg, Germany; 2 Division of Wildlife Ecology, Forest Research Institute of Baden-Württemberg, Freiburg, Germany; Universita degli Studi di Sassari, ITALY

## Abstract

Every year, there are millions of documented vehicle collisions involving cervids across Europe and North America. While temporal patterns in collision occurrence are relatively well described, few studies have targeted deer behaviour as a critical component of collision prevention. In this study, we investigated weekly and daily patterns in road crossing behaviour in roe deer. Using road crossing events and movement data obtained from GPS telemetry, we employed mixed-effect models to explain frequency and timing of crossings at five road segments by a number of predictors including traffic volume, deer movement activity and the presence of wildlife warning reflectors. We analysed 13,689 road crossing events by 32 study animals. Individual variation in crossing frequency was high but daily patterns in crossing events were highly consistent among animals. Variation in the intensity of movement activity on a daily and seasonal scale was the main driver of road crossing behaviour. The seasonal variation in crossing frequency reflected differences in movement activity throughout the reproductive cycle, while daily variation in the probability to cross exhibited a clear nocturnal emphasis and reflected crepuscular activity peaks. The frequency of road crossings increased as a function of road density in the home-range, while traffic volume only exerted marginal effects. Movement activity of roe deer in our study coincided with commuter traffic mainly in the early morning and late afternoon during winter and during periods of high spatial activity such as the rut. Both timing and frequency of crossing events remained unchanged in the presence of reflectors. Our results emphasise the importance of behavioural studies for understanding roe deer vehicle-collision patterns and thus provide important information for collision prevention. We suggest that mitigation of collision risk should focus on strategic seasonal measures and animal warning systems targeting drivers.

## Introduction

The ecological effects of roads and traffic on wildlife populations are a growing concern in many countries of the world (e.g., [1–4]), affecting essentially every moving terrestrial species [3–5]. Cervids *(Cervidae)* are heavily affected by roads through collisions with vehicles [6], with deer-vehicle collisions (DVCs) constituting the most important anthropogenic cause of mortality besides hunting in some regions [7]. Estimates of annual DVCs exceed one million deer in the USA and approximate one million in Europe [8,9]. The roe deer (*Capreolus capreolus*), a medium-sized ungulate, is a species especially prone to vehicle collisions. In Germany, for instance, approximately 200,000 roe deer are killed in collisions every year, thus accounting for most registered mammal-vehicle collisions in the country [10]. These collisions leave approximately 3,000 people injured and annually cause around 500 million Euro of property damage [10].

The probability of a DVC to occur is influenced by the relationship of three interacting factors: the frequency with which road segments are crossed by deer [11–13], the number and the speed of vehicles on this segment during a specific time period [14–16] and the characteristics of the road (e.g. roadside habitat; [17–19]). In practice, this relationship appears highly complex, with seasonally variable linkages between behaviour, the incidence of DVCs and the environmental attributes of a collision site (e.g. [14,20,21]). While road characteristics are fairly constant, both vehicle traffic and animal behaviour vary on a daily and seasonal scale [10,13,22]. Although a number of studies have addressed the role of road characteristics [18,19,23], only few have also analysed behavioural data of cervids in relation to roads (e.g. [19–21]) and none for roe deer. Hence, the temporal (i.e. daily, seasonal) variation in road crossing behaviour and its internal and environmental drivers remain largely unknown. This is relevant, because fluctuation in animal activity combined with variation in anthropogenic factors (e.g. traffic) create spatio-temporal risk zones for DVCs [20] and a better understanding may identify leverage points for collision prevention and provide critical knowledge for the evaluation of current prevention tools.

Conceptually, a road crossing attempt by a deer depends on (i) its behavioural state, namely a combination of its activity (i.e. intensity of movement activity; [20]) as well as the perceived reason for crossing the road at a given time (e.g. forage availability, migration; [14,21,24]), and (ii) the deterring effect of the road itself (i.e. mainly due to vehicle traffic, [24,25]). Accordingly, daily and seasonal differences in crossing occurrence should be related to variation in those parameters.

Roe deer show bimodal crepuscular activity peaks [26,27] and space use varies between seasons [28–30]. Analyses of temporal patterns in roe deer-vehicle collision occurrence have found visual similarities of temporal variation in collision incidence with the activity rhythm (e.g., [31–33]) and seasonal variation in movement activity (e.g., [7,8]). Most studies, however, did solely analyse collision data without analysis of roe deer behaviour. In this study, we explore how differences in roe deer behaviour (i.e. movement activity) relate to temporal variation in the occurrence of road crossings at a daily and seasonal scale. Based on the temporal patterns identified for DVCs, we predicted that both seasonal and daily variation in crossing occurrence reflect differences in roe deer movement activity throughout the day and between seasons.

In addition to movement activity, traffic is a particularly relevant influence for animals in roadside environments through noise, speed [19,34,35] and volume [23–25]. Cervids, in particular roe deer, possess the physiological capabilities required to perceive approaching vehicles and recognise them as a threat (e.g. to initiate evasive behaviour that prevents a collision; see [5]). As other species [13,24,25], roe deer may thus be expected to balance their need for crossing roads (e.g. to access resources) with the perceived risk of a road crossing and we predicted that periods of intense traffic would be characterised by fewer road crossings.

Finally, numerous collision prevention tools attempt to enhance the deterring effect of a road to induce a behavioural response in deer that reduces collision risk [9,36,37]. Of such, wildlife warning reflectors (WWRs) are among the most commonly used tools in both North America and Europe [38–40]. WWRs reflect the beam of a car’s headlights off or alongside the road during darkness based on the main assumption that animals respond to the stimulus presented by the illuminated reflector, causing animals to suspend or abandon the crossing attempt (see, [36,39,41]). In addition to such direct effects, the presence of prevention tools may influence collision risk by an adaptation of animal space use with regards to roads (i.e. for instance because at sites with a high traffic-density reflector devices may be continuously illuminated). An evaluation of potential long-term adaptations under the influence of prevention tools (i.e. shifts in road crossing frequency or time) remains elusive, because the behavioural foundation of road crossing occurrence is essentially unknown. We predicted that, in case WWR alter animal behaviour affecting road crossings by roe deer, the presence of WWR alters the frequency or the temporal distribution of road crossings at a site.

In this study, we used movement data of 32 GPS-collared roe deer in the vicinity of roads to investigate temporal patterns in road crossing events and their behavioural and environmental determinants. We studied seasonal and diurnal patterns in timing and frequency of road crossings and tested whether these were affected by i) variation in movement activity, ii) fluctuation in the volume of traffic at a site and iii) the presence of WWRs using an experimental before-after setting.

## Materials and methods

### Study area

**Fig 1:**
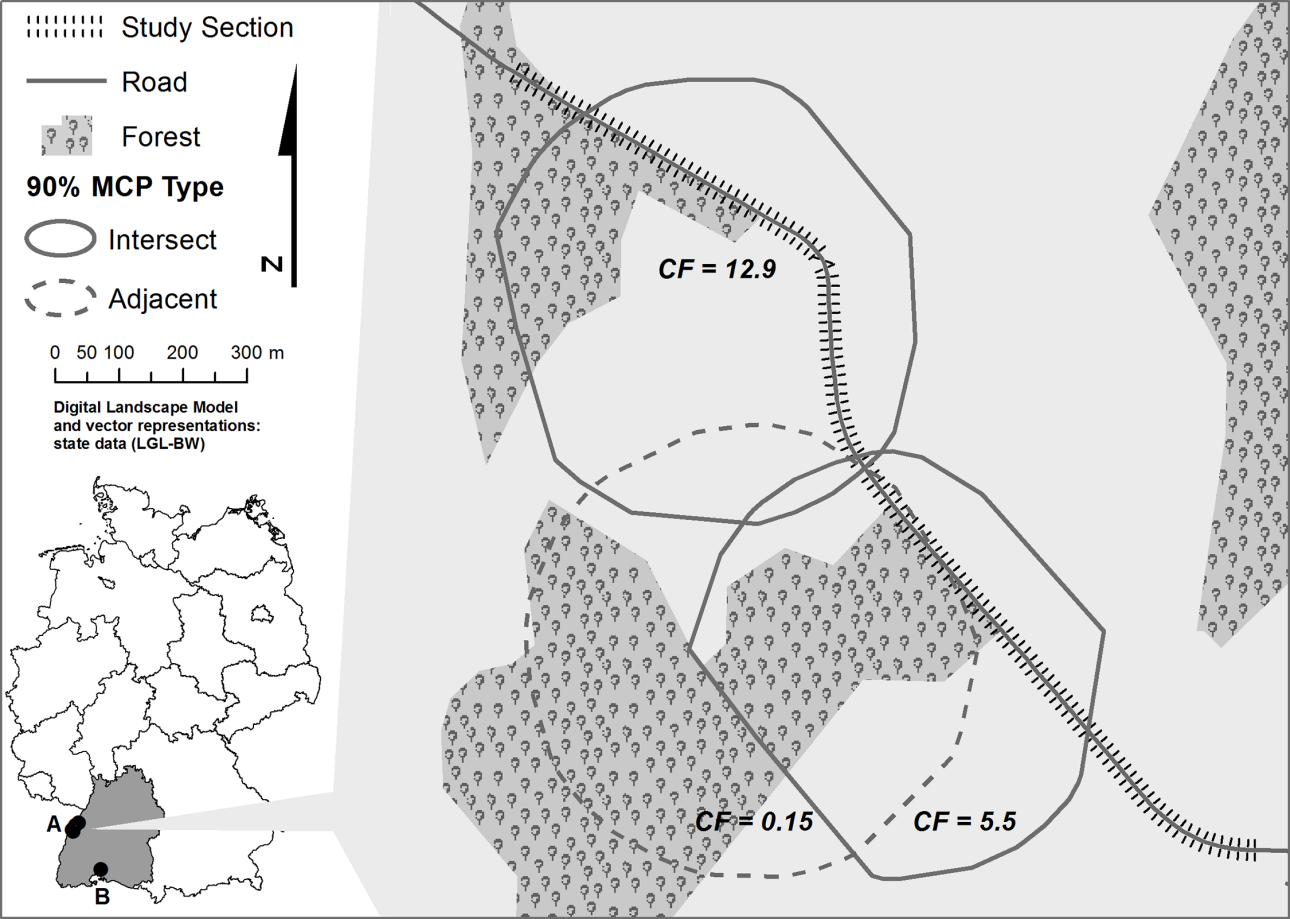
Conceptual illustration of the spatial layout of a study site in southwestern Germany. Four study sites were located in the Upper Rhine Valley (A) and another in the Hegau region (B). All sites were characterised by forest—open land mosaics and intermediary traffic volume. Exemplary weekly crossing frequencies (*CF*) are visualised for animals that utilised roads in their home ranges (HR) (HR allocation ‘intersect’) and those that used roads as HR boundaries (‘adjacent’).

The study took place in southwestern Germany (Fig 1). As study sites, we selected road sections that were characterised by high crossing frequencies and intermediate traffic volume to ensure frequent encounters between vehicles and deer. All road sections were surrounded by a mosaic of open (meadows, fields) and closed (forest) habitat features and possessed a minimum length of 200 m at a distance to urban settlements of >200 m. We only considered sites characterised by direct transitions of forest and agricultural land on either sides of a road as we expected those to be sites of high crossing occurrence and collision risk. Finally, they lacked features that could interfere with optimal reflector functioning: we selected sections featuring two lanes without median strip, level ground to both sides of the road, a straight course of the road and no guardrails, cycle lanes or steep slopes to either side of the road. Five study sites sharing this common spatial set-up (exemplified in Fig 1) met our criteria. Road sections were between 590 and 1,950 m in length (mean 1,420 m; S1 Table). Four of them were located in the Upper Rhine valley (N48.67, E8.00) at an altitude of 150 metres above sea level characterised by intensively farmed agricultural fields interspersed with forest stands. The fifth was situated in the Hegau region (N47.88, E8.73) at approximately 800 metres of altitude within a similar landscape configuration, but was characterised by a slightly higher proportion of forested area. The average number of vehicles per day at the study sites ranged from 1,035 to 3,856 (S1 Table).

### Data collection

At each study site roe deer were randomly caught in four consecutive winter seasons (October to March) between 2010 and 2013, using baited wooden box traps (size: 130 cm x 60 cm x 120 cm) with triggered release, drive nets or up nets. All capture, tagging, and monitoring protocols were approved and permitted by the animal welfare and hunting administration of the federal state of Baden-Württemberg, Germany (Regierungspräsidium Freiburg; G-09/53). All deer that weighed more than 15 kg and showed good health were equipped with GPS tracking collars. Collars were produced by e-obs GmbH, Germany, and weighed 370 g (lifetime approx. 50,000 GPS fixes; accuracy approx. 10 m).

In total, 46 animals, 17 males and 29 females, were equipped with tracking collars. GPS-locations were collected with an acceleration-informed GPS-schedule [42] at a 15-minute fix interval when deer were active, and at a 180-minute fix interval once the variance of five consecutive acceleration-bursts of the z-axis was below a threshold of 1,000. Traffic data (i.e. number of vehicles and vehicle speed) were collected for all sites and both lanes during several periods between 2011 and 2014 using high-performance traffic data classifiers (‘SDR Traffic’; DataCollect Traffic Systems GmbH, Germany). The traffic data classifiers were mounted at a height of 1.2 m on steel posts at 1 m distance to the road. Traffic data were collected continuously throughout the day.

Between July 2012 and March 2013, WWRs consisting of a black plastic semi-cylinder covered in blue reflector foil (3M foil Type RA3; Schilderwerk Beutha GmbH, Germany; S1 Fig) were placed on delineators at a distance of 50 metres along both sides of the road at each study site. Reflectors of this type reflect the light of a car’s headlights along the side of the road back to the light source(see [39]). WWRs were installed approximately halfway into the study period (S1 Table), with treatment and control phases spanning at least one year each to control for confounding seasonal variation. The characteristics of the study sites determined the spatial extent of reflector placement.

### Data processing

All analyses were performed in R [43] and data handling was carried out in Coordinated Universal Time (UTC). Local time was converted to UTC prior to analysis. Road crossings by deer were defined as the linear intersections of the lines connecting two consecutive GPS fixes and a road. We removed inaccurate GPS-fixes and thus potentially false crossing events by filtering GPS-locations using a measure of horizontal GPS-fix accuracy provided by the collar. We filtered the remainder of the dataset to include only those study animals, which consistently occupied a home range (i.e. non-dispersing deer) that included or bordered onto a road section fitted with WWRs to ensure that animals had physical access to the road. Home ranges (HR) were calculated using 90% minimum-convex-polygons (MCP) as these provided an adequate representation of individual roe deer space use at our study sites. To account for potential differences in crossing occurrence due to roads cutting through the centre rather than the fringes of a home-range, we classified home-rage location with regards to roads into a categorical variable, thus separating animals whose home-range boundaries were oriented along road segments from those whose HR included the road (henceforth: variable *HR allocation;* see Fig 1). To exclude an underlying effect of home-range composition on home-range allocation relative to roads we visually compared habitat composition among animals and tested for differences in the percentage of forest cover and home-range size between the two groups.

We targeted temporal patterns of road crossing occurrence on two temporal scales. On a seasonal scale, we focused on factors influencing the weekly frequency of road crossings and investigated whether the placement of WWRs caused a decrease in overall frequency. In a second step, we targeted the probability of a crossing to occur on a daily scale in dependence of movement activity and traffic volume and investigated whether the placement of WWRs would affect the probability and timing of road crossing events.

### Road crossing frequency

To assess seasonal patterns in road crossings we pooled crossing events per individual into a count of road crossings per week and study animal. We assigned zeros to all weeks during which no road crossing event was recorded. Spatial behaviour of both sexes was expected to vary between seasons [44–46], especially in relation to reproduction. We thus obtained the mean number of daylight hours for each week to account for seasonality (henceforth: *day****-****length*). We calculated the extent of road sections equipped with WWRs within the home-range of each study animal and normalised section length by the home-range size as a proxy for exposure to the treatment (as metres per ha of HR size; henceforth: *road exposure*). Finally, we obtained the total length of all consecutive line segments between GPS fixes of tagged roe deer for each week in the dataset and for each study animal as a proxy for the intensity of roe deer movement activity.

### Road crossing occurrence

To study daily patterns in the occurrence of crossing events, we assigned each crossing to the respective hour of the day at which it occurred (as 0 to 23). We then created a new dataset consisting of 24 observations per day (i.e. 24 hours) for each day on which a crossing event was recorded for each respective study animal. All hours during which a crossing was observed received value 1, while the remainder was assigned zero and served as unused units in our analysis. We were confident that potential bias from false negatives is negligible due to the high temporal resolution of the data (ΔT ~ 15 min) and the size of the dataset (>10,000 hours with a crossing event). We then assigned the daily number of daylight hours to each record as a measure for seasonality and aggregated the length of all consecutive line segments between GPS fixes in each hour in the dataset for each study animal as a measure of movement activity. In addition, traffic volume was calculated as the sum of cars at the site during each hour. Our traffic dataset did not cover the whole study period. We therefore obtained average traffic volume for all hours of each weekday (Monday to Sunday) and each study site and used these to supplement the missing data. Traffic volume was averaged separately for winter and summer time (i.e. daylight saving time), in order to avoid systematic bias from UTC time conversion. We considered this practice justified, because a preliminary analysis revealed site- and weekday-specific differences in traffic volume, but daily variation was otherwise highly consistent throughout the year. We verified that traffic volume remained constant after the placement of WWRs at all sites. We classified each observation to have taken place either during the night (darkness) or during the day (light). Hours were classified as dark if more than 50% of an hour took place before (morning) or after (evening) civil twilight. Civil twilight times were calculated with the library maptools [47] using a sun angle of 8 degrees under the horizon.

### Statistical analyses

#### Road crossing frequency

We used Linear Mixed Models (LMMs, using package nlme; [48]) to assess seasonal patterns in weekly road crossing frequency related to movement activity and the location of the home-range relative to a road. The number of weekly road crossings was square root transformed to meet parametric assumptions regarding normality of residuals. The software implementation in R gave us greater flexibility in accounting for individual behaviour and non-independence of our data when fitting a LMM compared to a GLMM. Individual differences between study animals were accounted for by fitting random intercepts for each study animal ID nested within study sites. We additionally incorporated individual differences in road crossing behaviour by allowing for heterogeneous variances for each study animal in the model. Candidate model structures were compared using Akaike’s corrected Information criterion (AICc). Temporal autocorrelation among subsequent weeks was addressed by including an autocorrelation structure, modelling a decreasing degree of autocorrelation with increasing temporal distance between data points. We chose an autoregressive moving average (ARMA 1,1) covariance type for individual study animals at each site as this resulted in the best fit. After setting the random-effect structure of the model, we employed a hypothesis driven approach in model selection. We specified our fixed effect structure based on our hypotheses, because we were interested in the effects of a particular set of predictors on road crossing behaviour in roe deer. Our final model featured the distance covered each week by each animal (range 804–43,397 m), the HR allocation (factor: adjacent vs. intersect), the road exposure (in metre / ha HR; range 12.8–37.1), the mean weakly day-length (range 8–16 hours) and the presence of WWRs (factor: absent vs. present). We included a quadratic effect for the distance covered each week by each animal to allow for a non-linear relationship of deer movement activity and crossing occurrence.

#### Road crossing probability

We used generalised linear mixed-effect models (GLMM, using package lme4; [49]) to assess daily patterns in the probability of a road crossing to occur in relation to movement activity and traffic volume at a site. We employed a GLMM with binomial error and logit link. As response we used the hours during which crossings were recorded for each animal contrasted to hours during which no crossing was recorded (a similar approach has been used by Thurfjell et al. [13] for the analysis of collision data). The dataset was accordingly treated as used-unused data. We included animal ID nested within study site ID to account for study design. We also included crossed random intercepts for the year of the study into the model. Again, we specified our fixed effects in accordance with our hypotheses. The distance moved during each hour (range: 0–2,658 m) and its quadratic effect, the hourly traffic volume (range: 1–1,458 cars) and its quadratic effect, the variable indicating darkness vs. daylight, the day-length and the presence of WWRs were included as predictors. We also included interactions of both day-length and reflector presence with the predictor representing day vs. night as biologically meaningful.

All continuous predictors were scaled by the standard deviation and centred to zero in both models to allow for comparison of effect sizes. Parametric assumptions for the use of a linear and generalised linear mixed model were met by our final models, respectively. Final model coefficients were estimated using restricted maximum likelihood (REML). We calculated the conditional and marginal coefficient of determination for mixed-effect models to assess absolute model quality for both of our models using r.squared GLMM in MuMIn [50,51].

## Results

Our final dataset comprised 13,689 road crossing events of 32 study animals that contributed data over variable periods between February 2010 and October 2014. There were large individual differences in the amount of recorded road crossing events (overall mean = 409, SD = 508; weekly mean = 6.1, SD = 5.5; see S2 Fig). We recorded at least one crossing event on 99.8% of all experimental days; our analysis was therefore not influenced by periods of seasonally missing data. In general, we could distinguish two types of deer in our dataset with regards to second-order habitat selection. The majority of animals utilised roadside environments and roads therefore intercepted their HR (N = 27). The remaining animals (N = 5) used roads as home-range boundaries. Although the home-ranges of the latter type were typically completely devoid of roads, we found no differences in home-range size and composition (Mann-Whitney-U test forest cover: W = 0.69, p = 0.788; HR size: W = 105, p = 0.143).

### Road crossing frequency

**Fig 2:**
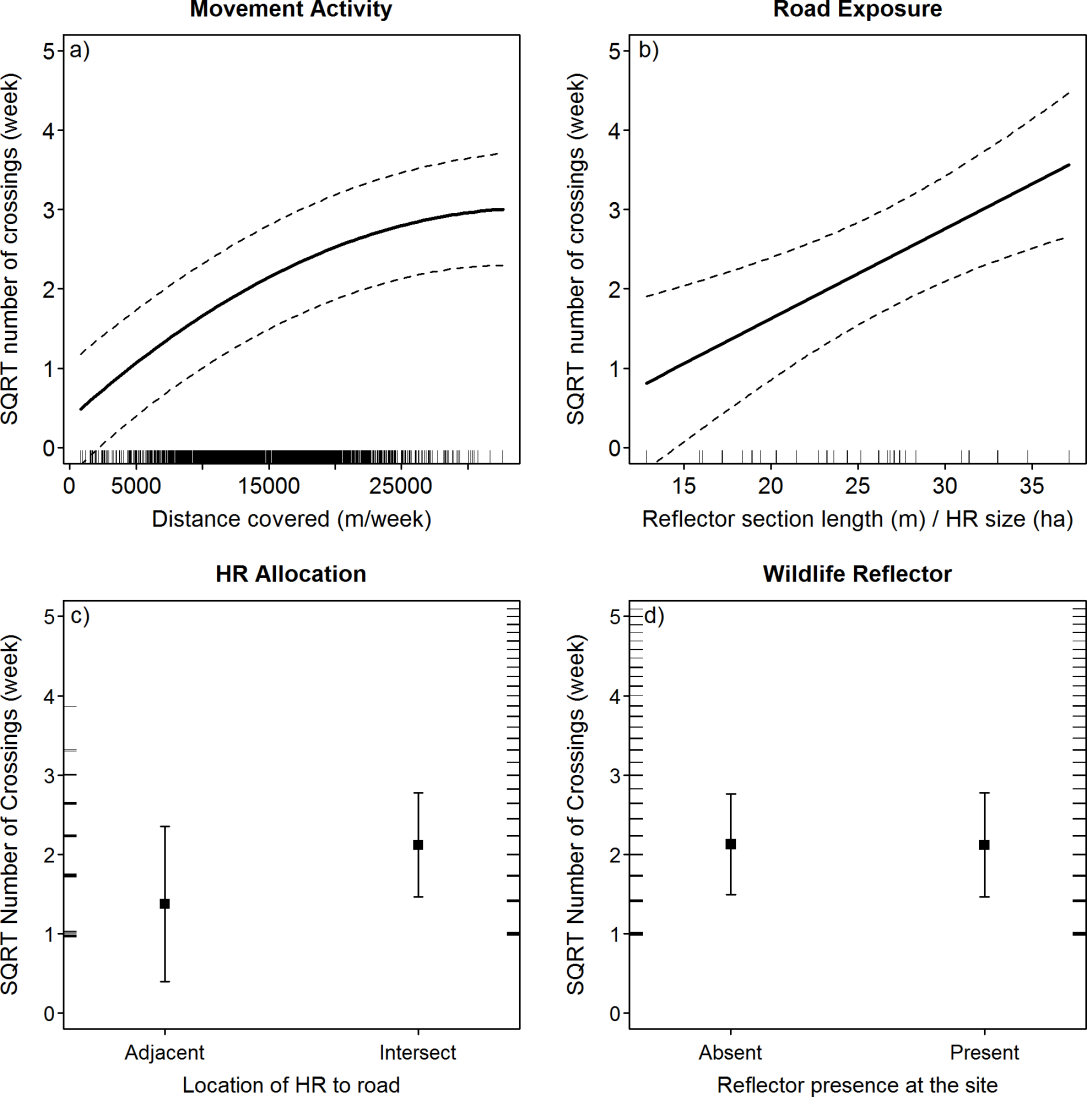
**Predicted variation in the amount of road crossings under the influence of a) movement activity, b) the degree of exposure to a road, c) the allocation of the HR in relation to the road and d) the presence of wildlife warning reflectors at the sites.** Note that weekly crossing frequencies were square-root-transformed. Dotted lines denote 95% confidence intervals over the fixed effects. Predictions were obtained with continuous covariates set to the median.

**Fig 3:**
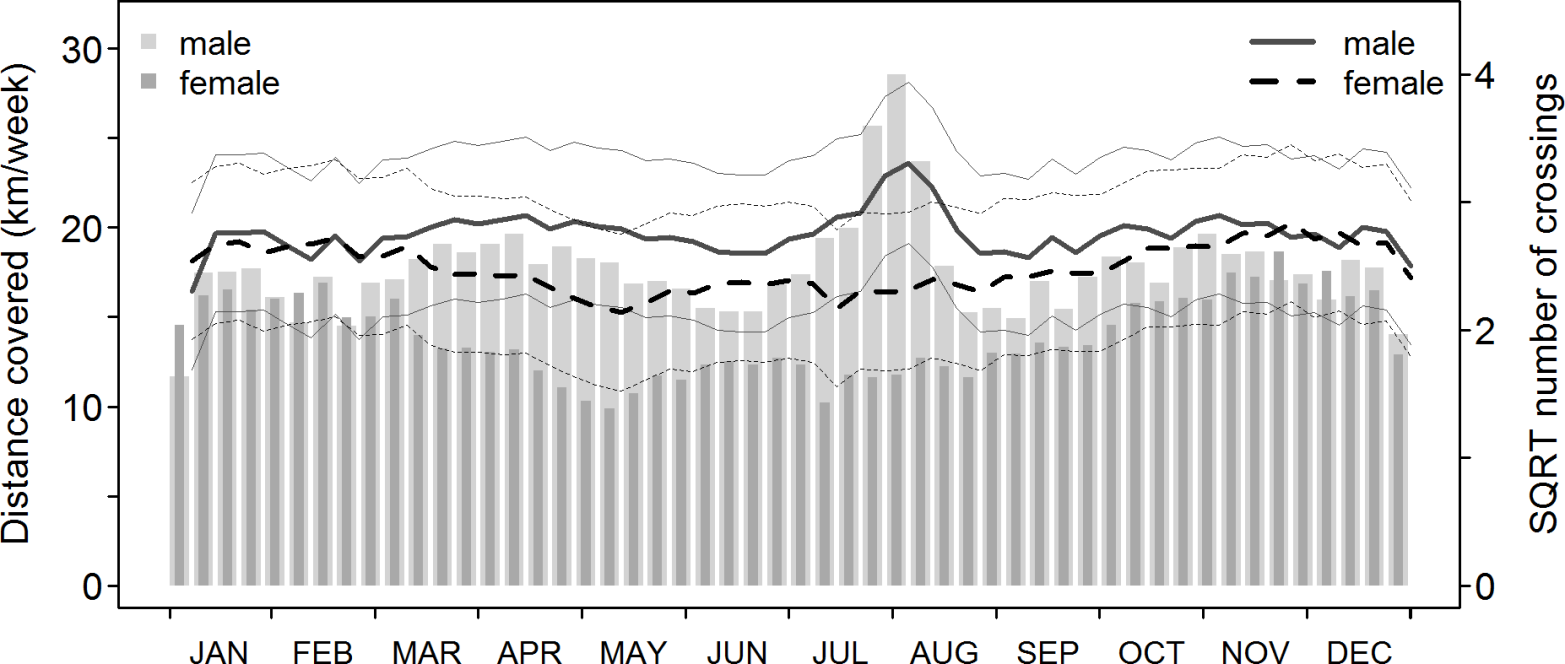
Annual variation of movement activity and the predicted road crossing occurrence for animals of intersecting home-range type (i.e. roads included in the home-range). Bars depict variation in movement activity for male and female deer throughout the year. Bold lines show the corresponding number of weekly road crossings predicted by the large-scale model (for animals of type ‘intersect’) and thin lines indicate 95% confidence intervals over the fixed effects. Predictions were obtained with continuous covariates set to the median.

We found the movement activity of animals to be a key driver of weekly road crossing frequency, with weekly crossing numbers increasing with the total distance moved throughout each week (p < 0.001, Table 1; Figs 2A and 3; raw data in S1 Data). Overall, males exhibited a higher movement activity than females, especially during the rut (Fig 3). Female movement activity decreased around the time of parturition, but this was only weakly reflected in the predicted amount of road crossings (Fig 3). The number of crossings increased as a function of the length of road within the HR (p = 0.002, Table 1; Fig 2B) and crossing frequencies were lower in animals that used roads as HR boundaries, but not significantly so (p = 0.196, Table 1; Fig 2C). In addition, there was a weak seasonal effect, with more crossings occurring on days with a higher number of daylight hours (p = 0.038, Table 1). We found no evidence for an effect of the reflector devices on the frequency of road crossings (p = 0.991, Table 1, Fig 2D; predicted difference in mean number of weekly road crossings after reflector placement ΔF = 0.009). The fixed effects in our model only explained a limited part of the variability in the data (marginal R^2^; = 0.28), while the whole model explained a considerably larger part (conditional R^2^; = 0.50), indicating strong behavioural plasticity at the individual level. The variance of the random intercepts was estimated at 0.12 around the intercept of 0.643 (range of individual intercepts: -0.59 to 0.95).

**Table 1:** Final model results for weekly crossing frequencies (CF). Coefficients (ß), coefficient standard errors of all predictors (SE (ß)) and p-values are provided. The response variable was square-root-transformed. All predictors were standardised to allow for comparison of effect sizes. Variables with a p-value < = 0.05 are highlighted *italic*.

*CF*		*IC*	RP	*DIST*	*DIST^2^;*	*SHR*	TP	*DL*
**√x**	**β**	1.314	-0.002	0.504	-0.072	0.678	0.721	0.122
	**SE(β)**	0.508	0.150	0.038	0.014	0.201	0.543	0.059
	**p**	0.01	0.991	0.000	0.000	0.002	0.196	0.038

*IC*: Model intercept, *RP*: reflectors present at the sites (reference: WWR absent), *DIST*: distance covered per week, *SHR*: road exposure as reflector section length per ha of HR, *TP*: home-range type being adjacent to road (reference: intersect), *DL*: Day-length on each day, β: model parameter estimate, *SE* (β) standard error of the model beta.

### Road crossing probability

**Fig 4:**
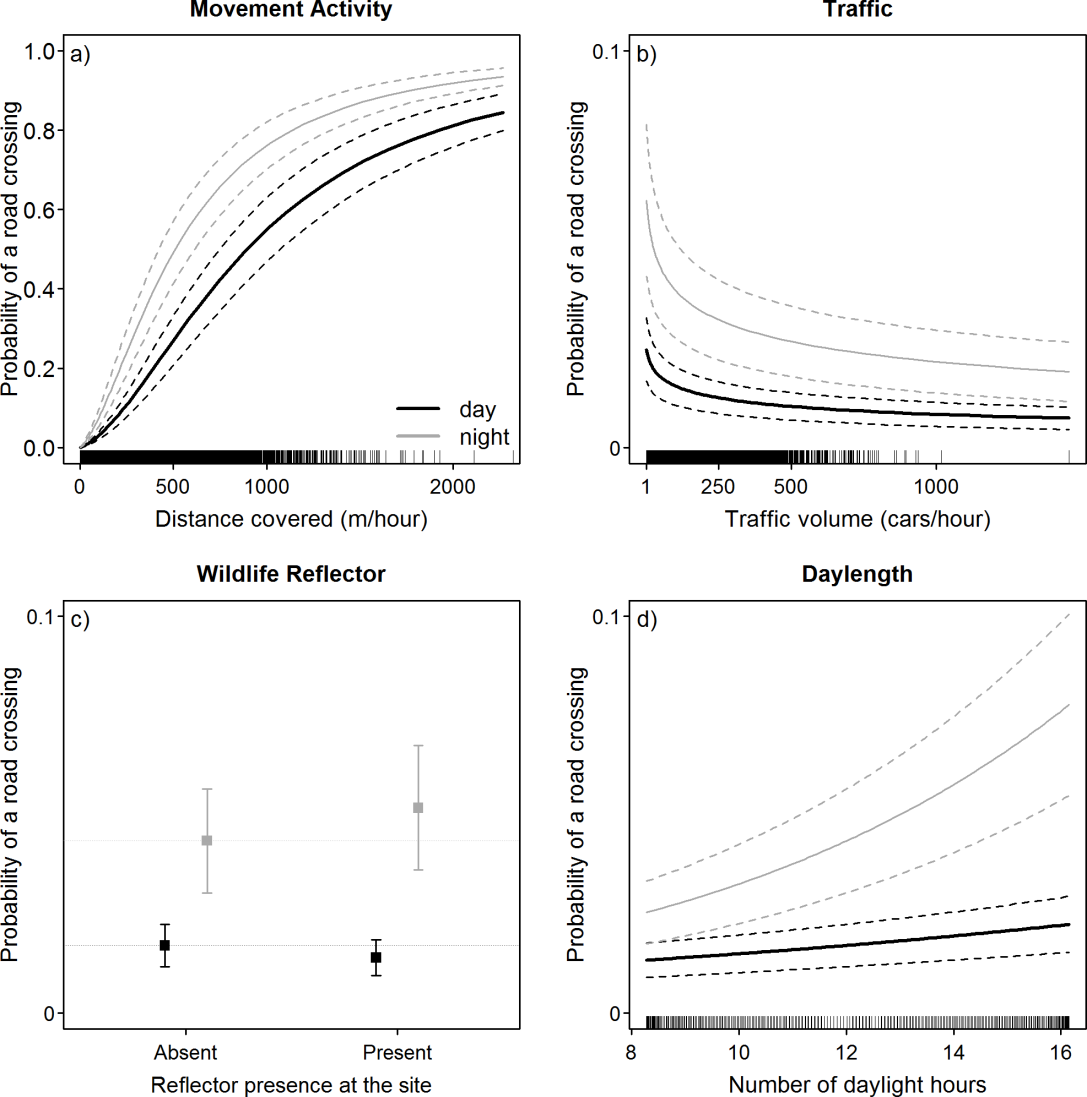
**Predicted probability of road crossings to occur for day (black line) and night (grey line) dependent on a) movement activity, b) the traffic volume, c) the presence of wildlife warning reflectors at the sites and d) the variation in the amount of daylight hours (i.e. day-length).** Dotted lines denote 95% confidence intervals over the fixed effects. Predictions were obtained with continuous covariates set to the median.

**Fig 5:**
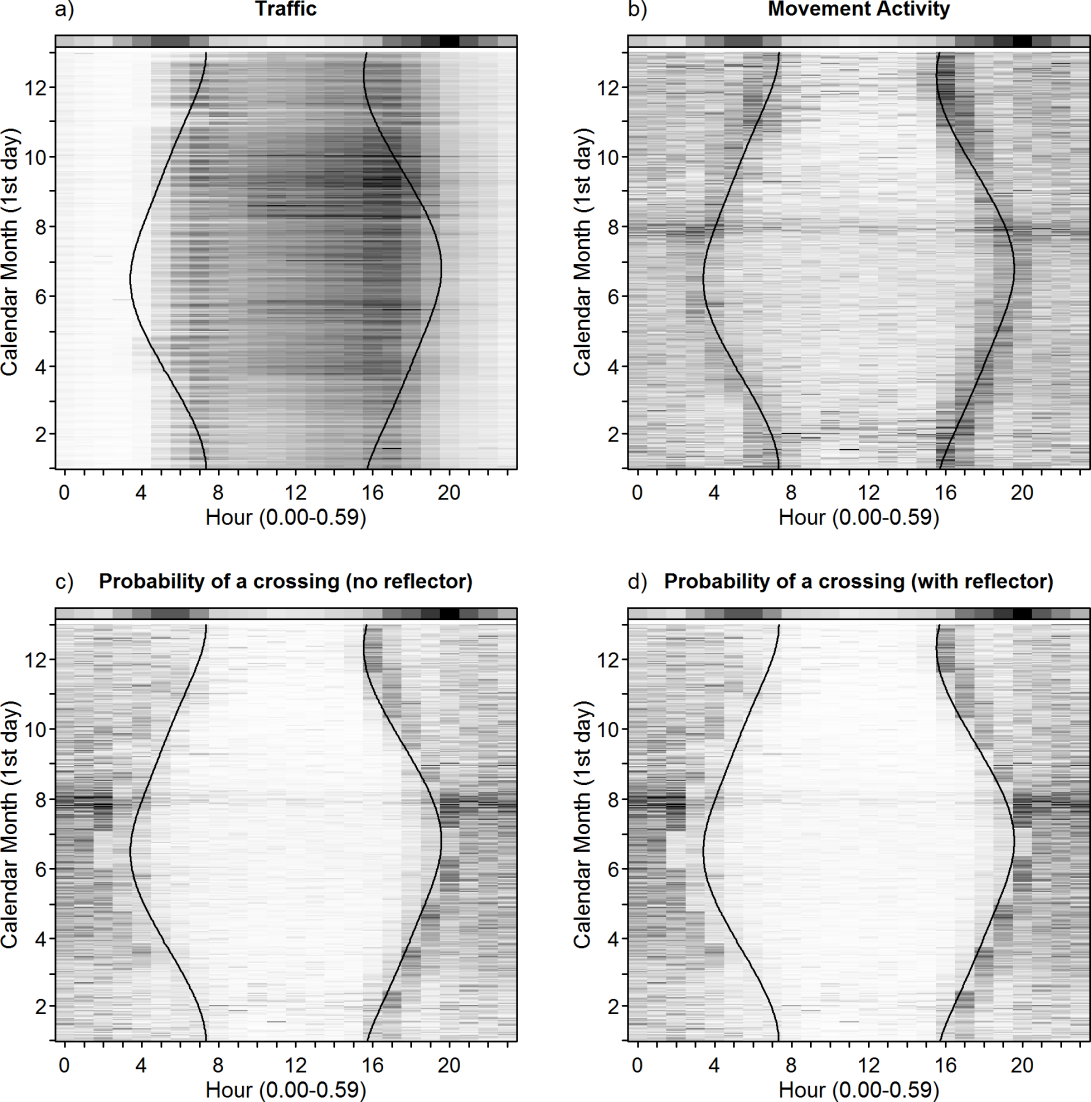
**Temporal variation (daily and annual) in a) mean traffic volume (range: 1–355 vehicles/h), b) mean movement activity of the animals (range: 3–550 m/h) and the probability of a road crossing to occur as predicted by the model in c) the presence and d) absence of wildlife warning reflectors.** Darker values indicate increasingly high values and *vice versa*. Note that traffic and movement activity were averaged across all studied animals in this figure. The colour bar at the top of each panel denotes the diurnal pattern of roe deer vehicle collisions reported by Steiner et al. [7] and four references therein (as percent DVC occurrence for each hour of the day). The black lines indicate the variation in the time of sunrise and sunset throughout the year.

Movement activity was likewise the main determinant of hourly crossing occurrence. The probability of a crossing to occur increased with the distance moved within one hour (p < 0.001, Table 2; Fig 4A; raw data in S2 Data). There was a weak effect of traffic volume (p <0.001, Table 2) and particularly during twilight hours, reflecting patterns in deer movement activity (Fig 5). Nocturnal road crossings were slightly more likely to occur on days with a higher number of daylight hours (Fig 4D). Finally, differences in the probability of a crossing to occur after the placement of WWRs were negligible and associated effect sizes were small (Table 2; predicted change in mean crossing probability: for daytime Δp = 0.003 and night-time Δp = 0.008; see Fig 4C) although p-values indicated significance (p < 0.001, Table 2). The final model explained a considerable part of the variability in the data, with inter-individual variation being less important in explaining the diurnal variation in crossing events than for the seasonal variation in frequency (marginal R^2^; = 0.54; conditional R^2^; = 0.57). The variance of the random intercepts was estimated at 0.11 around the intercept of -4.264 (individual range: -0.82 to 0.57).

**Table 2:** Final model results for the analysis of road crossing probabilities (CP). Model coefficients (ß), coefficient standard errors of all predictors (SE (ß)) and p-values (p) are provided. All predictors were standardised to allow for comparison of effect sizes. Variables with a p-value < = 0.05 are highlighted *italic*.

*CP*		*IC*	*RP*	*DIST*	*DIST^2^;*	*DN*	*DL*	*TVOL*	*TVOL^2^;*	*DN*:*DL*	*DN*:*RP*
**0–1**	**β**	-4.264	-0.200	1.968	0.180	0.974	0.177	-0.232	-0.040	0.219	0.383
	**SE(β)**	0.163	0.054	0.040	0.026	0.036	0.024	0.019	0.013	0.028	0.060
	**p**	0.000	0.000	0.000	0.000	0.000	0.000	0.000	0.001	0.000	0.000

*IC*: Model intercept, *RP*: reflectors present at the sites (reference: WWR absent), *DIST*: distance covered in each hour, *DN*: day or night (factor, reference level: day), *DL*: day-length on each day, *TVOL*: traffic volume for each hour (sum of cars), β: model parameter estimate, *SE* (β): standard error of the model beta.

## Discussion

We found temporal patterns in road crossing events to closely reflect variation in movement activity of roe deer. In accordance with our expectations, the weekly frequency of road crossings was clearly affected by seasonal differences in movement activity that were associated with the reproductive cycle of roe deer. In our study, males exhibited overall higher levels of movement activity than females, with a distinct peak in males’ movement activity during the rut, and a marked low activity of females during the period after parturition (see Fig 3). As hypothesised, the daily variation in the probability of a road crossing to occur likewise reflected patterns in movement activity of roe deer. We found road crossings to occur primarily at night and infrequently during the day (Fig 5C and 5D). The bimodal crepuscular activity pattern previously described for roe deer (e.g., [26]) was clearly reflected by the movement activity in our study (Fig 5B). Crepuscular activity peaks were associated with peaks of high crossing probability during dawn and dusk (Fig 5), coinciding with the commonly reported collision peaks during twilight hours (e.g., [7,31,33,52]; Fig 5). The results of our study thus indicate that road crossings are mainly driven by the behavioural patterns of roe deer rather than directly by the volume of traffic on a road (i.e. in contrast to our expectations; Fig 4B and 4C). Animals at our study sites were continuously exposed to vehicles and may thus be expected to exhibit a certain degree of habituation to this stimulus. Although coinciding with overall low traffic volumes, the higher probability for a nocturnal crossing event likely reflects a concentration of movement activity within those hours characterised by fewer anthropogenic stressors rather than a direct behavioural reaction to traffic. This assumption is supported by the marginal effect of traffic volume in our model and the overlap of crossing behaviour and traffic patterns on days with shorter daylight (compare Fig 5A and 5C).

With regards to seasonality in daily crossing patterns, our results show that animals respond to a changing day-length by accentuating their crossing behaviour into darkness hours as the day-length increases, while crossings occur further into the afternoon and the early morning on days with short daylight (i.e. in winter when darkness falls earlier). As a consequence, crossing occurrence overlaps with commuter traffic peaks early in the day and late in the afternoon, especially on days with short daylight. Twilight peaks in collision occurrence are thus likely a reflection of this overlap, since collision risk must be expected to be low during the other hours of the day when crossing occurrence and traffic intensity hardly overlap (see Fig 5). This is in agreement with the daily pattern in collision occurrence described in a review by Steiner et al. [7], which is included for comparison in Fig 5. On a seasonal scale, the link of movement activity with crossing frequency also suggests a relationship with the annual variation in collision risk. The seasonal pattern in the incidence of roe deer-vehicle collisions appears heterogeneous across Europe (see Steiner et al. [7] for a comprehensive review). A common pattern reported by some authors exhibits peaks in the spring and early summer, i.e. particularly between April and June, and to a lesser extent during the rut (e.g., [31,53,54]). This pattern appears especially pronounced for males (e.g., [53,54]). In the absence of official collision records, accounts of roe deer road mortalities at our sites confirm an April peak in general collision risk (S3 Fig, unpublished data). However, other authors have found a rather homogenous seasonal distribution of DVC [7,54]). Rodríguez-Morales et al. [31] and Pokorny [52] relate the spring peak to pre-birth displacement of adult females, dispersal of (male) yearlings and territorial behaviour of adult males. Additionally, more intense foraging activity of lactating females might play a role [31]. This is consistent with the predictions of our model that periods of higher movement activity (e.g. the rut) result in higher road crossing frequencies and thus in a potentially higher risk of roe deer-vehicle collisions. The results of our study do, however, only apply to patterns of road crossing occurrence in resident individuals. Accordingly, our models predict a peak in collision risk during July and August where movement activity is most intense, which is supported by patterns of vehicle collisions involving our studied animals, of which 35% (6 of 17) occurred in July and only 2 of 17 in April-Mai (see S4 Fig, unpublished data).

We could confirm that roe deer, which were exposed to longer road sections (i.e. relative to the home-range size) crossed roads more frequently. However, we identified two distinct patterns in second-order resource selection by roe deer. Some individuals used roads as HR boundaries and accordingly crossed roads rarely, whereas the HR of other individuals included roads and their crossing frequency reflected exposure to the road. Regardless, strategies of deer towards roads are likely a behavioural gradient. This is supported by the strong inter-individual differences in the amount of recorded crossings which are also reflected by the importance of the random term in our model of weekly crossing frequency. These results are in accordance with the behavioural plasticity that has been reported for the species (e.g., [7,28,55]).

In contrast to our expectations, the timing and frequency of road crossings remained unchanged after the placement of WWRs at our study sites. Although WWRs are primarily designed to momentarily suspend a crossing attempt so that road crossings occur during periods when reflectors are not illuminated (i.e. no traffic on the road), we hypothesised that, if the additional stimulus provided by WWR presence would affect crossing attempts directly, this effect may also lead to changes in crossing frequency or a temporal shift in crossing attempts (e.g., to times of low traffic volume). However, the predicted differences in both road crossing frequency and the probability of a road crossing to occur were marginal, with essentially no difference in road crossing frequency (i.e. the equivalent of approximately 0.5 road crossings per animal per year; see also Fig 2D) and a reflector-induced change in the mean daily probability of a crossing of < 0.01 (see Fig 4C). Even though the resolution of our data does not permit an assessment of roe deer behaviour during individual crossing events, unchanged crossing frequencies in the presence of reflectors indicate that WWRs did not increase the barrier effect of roads. In principle, this is a desirable property for a collision prevention device if individual crossing events could be sufficiently influenced to lower collision risk while retaining the permeability of the road for animal movement. However, studies on direct behavioural effects of WWRs have demonstrated that reflectors did not significantly alter deer behaviour (white-tailed deer *(Odocoileus virginianus)*: [40,56], fallow deer *(Dama dama)*: [57]). Furthermore, a comprehensive reanalysis of animal-vehicle collision data based on 43 studies revealed no significant reduction in collision rates after placement of light reflecting devices [39]. We suppose that a reduction of roe deer-vehicle collisions, as reported by some authors [58–60] and practitioners, may be due to alterations in drivers’ behaviour induced by the reflectors (e.g., higher awareness due to the additional stimulus leading to lower collision risk). Although this aspect was already mentioned by Zacks in 1986 [61], we are not aware of any study investigating drivers’ responses to the presence of WWR.

### Practical implications

Our results demonstrate the importance of animal behaviour as a crucial component of collision risk, which is especially evident for the temporal variation in road crossing behaviour. Based on the temporal variation in roe deer movement activity in our study, the occurrence of crossing events has the greatest overlap with peaks in commuter traffic in the early morning and late afternoon, especially during winter months (i.e. October—March), but also throughout the day during periods of high spatial activity such as the summer months (i.e. July, August). However, our findings only reflect risk patterns associated to non-dispersing deer. The study provides a valuable baseline for the design of mitigation measures targeting collisions with roe deer in cultural landscapes.

In general, we suggest that the manipulation of drivers’ behaviour in conjunction with an alteration of animal behaviour bears the greatest potential to be pursued. We suggest the implementation of flexible mitigation measures featuring a strategic seasonal component, such as e.g. seasonal speed limits during particular times of the day or animal detection systems attempting to modify drivers’ behaviour. Although we did not confirm an effect of wildlife warning reflectors on frequency and timing of roe deer road crossings, the application of particular types of illumination has been found to induce a behavioural response in free-ranging white-tailed deer [62] and captive roe deer [63]. Therefore, we suggest further exploration of the potential of light as a tool in collision prevention (e.g. additional light sources in vehicles), especially for species that are heavily involved in vehicle collisions worldwide.

## Supporting information

S1 Table Characteristics of the study sites. The amount of animals captured at all sites is provided along with the length of the road segment along which reflectors have been placed (in meters), the date of reflector placement and the daily traffic volume (as vehicles per day).
(DOCX)

S1 Fig Wildlife warning reflector produced by Schilderwerk Beutha GmbH, Stollberg, Germany. Reflectors consist of a black plastic semi-cylinder covered in blue reflector foil (3M foil Type RA3). Reflectors are mounted on delineators, which are set up with 50 m spacing and at a distance of 50 cm to the road.
(DOCX)

S2 Fig Overview of inter-individual variability in weekly crossing frequencies. Boxplots depict variation in the amount of recorded road crossings per week for each study animal. Numbers in the top row denote the amount of weeks that each animal provided data (N). Black bars indicate the median of road crossing frequency for each animal and boxes delimit the interquartile range. Colours are arbitrary.
(DOCX)

S3 Fig Pooled roe deer-vehicle collision records by month over the duration of the study. Collision records were available for three out of five road sections used in the study.
(DOCX)

S4 Fig Overview of confirmed roe deer-vehicle collision involving GPS-collared studied animals (N = 17 of 46); males light grey, females grey. In addition, another 14 studied animals were found dead, but the cause of death was unclear.
(DOCX)

S1 Data (TXT)

S2 Data (ZIP)
