# Supplementary material for: Temporal patterns in road crossing behaviour in roe deer (Capreolus capreolus) at sites with wildlife warning reflectors
Source: PLoS One. 2017 Sep 27;12(9):e0184761. doi: 10.1371/journal.pone.0184761 (PMC5617160; doi:10.1371/journal.pone.0184761)
Supplement: S1 Table — The amount of animals captured at all sites is provided along with the length of the road segment along which reflectors have been placed (in meters), the date of reflector placement and the daily traffic volume (as vehicles per day). (DOCX) [file pone.0184761.s001.docx]

**S1 Table. Characteristics of the study sites.** The amount of animals captured at all sites is providedalong with the length of the road section along which reflectors have been placed (in metres), the date of reflector placement andthe daily traffic volume (as vehicles per day).

| Study Site | No. Animals | Males | Females | Section length | WWR date | Traffic volume |
| --- | --- | --- | --- | --- | --- | --- |
| *Oberbruch* | 5 | 1 | 4 | 590 m | 04.07.2012 | ~4000 / day |
| *Gamshurst* | 11 | 2 | 9 | 1780 m | 04.07.2012 | ~2000 / day |
| *Zierolshofen* | 9 | 5 | 4 | 1950 m | 11.01.2013 | ~1000 / day |
| *Sand* | 9 | 4 | 5 | 1910 m | 24.01.2013 | ~2000 / day |
| *Stetten* | 12 | 5 | 7 | 870 m | 05.03.2013 | ~2000 / day |
| Total | 46 | 17 | 29 | - | - | - |
