# Supplementary material for: Temporal patterns in road crossing behaviour in roe deer (Capreolus capreolus) at sites with wildlife warning reflectors
Source: PLoS One. 2017 Sep 27;12(9):e0184761. doi: 10.1371/journal.pone.0184761 (PMC5617160; doi:10.1371/journal.pone.0184761)
Supplement: S1 Fig — Reflectors consist of a black plastic semi-cylinder covered in blue reflector foil (3M foil Type RA3). Reflectors are mounted on delineators, which are set up with 50 m spacing and at a distance of 50 cm to the road. (DOCX) [file pone.0184761.s002.docx]

| 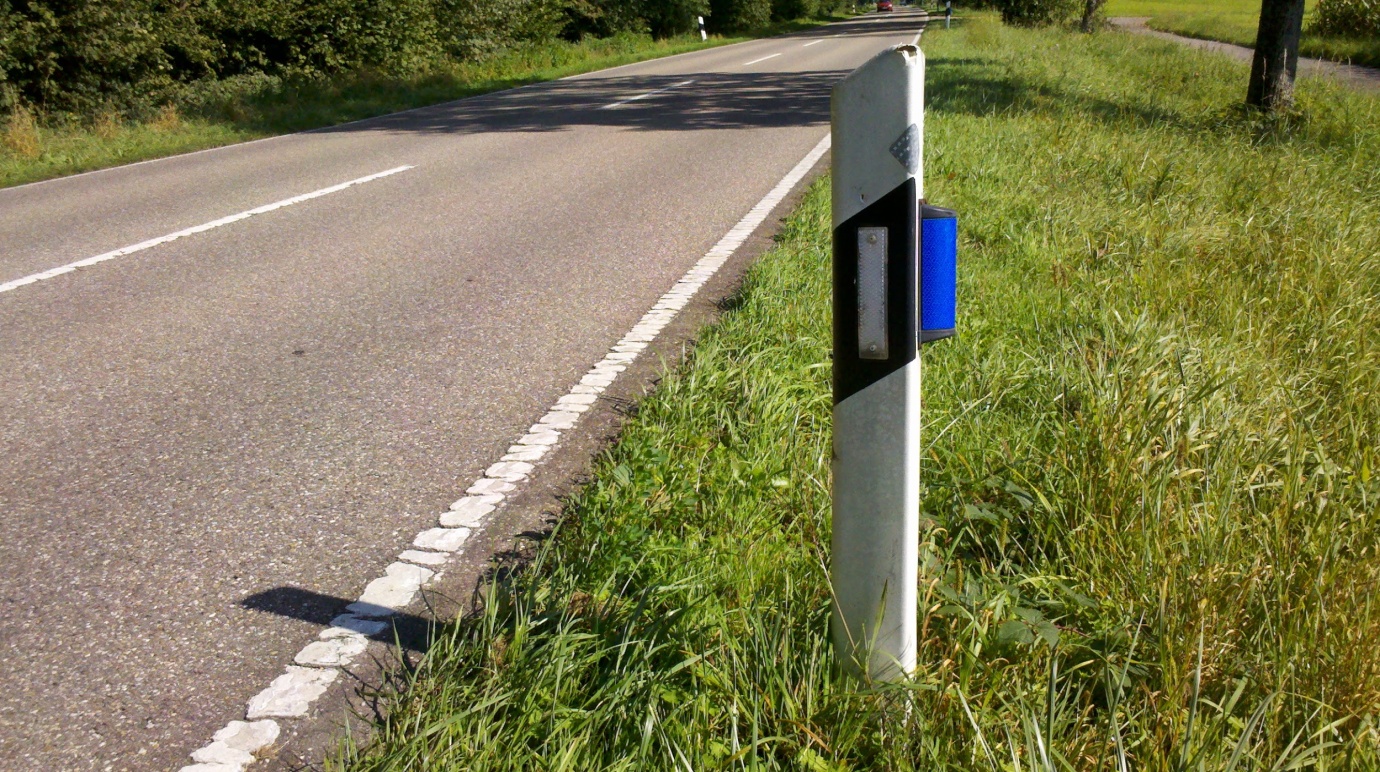 |
| --- |
| **S1 Fig. Wildlife warning reflector produced by Schilderwerk Beutha GmbH, Stollberg, Germany.** Reflectors consist of a black plastic semi-cylinder covered in blue reflector foil (3M foil Type RA3). Reflectors are mounted on guideposts, which are set up with 50 m spacing and at a distance of 50 cm to the road. |
