# Supplementary material for: Temporal patterns in road crossing behaviour in roe deer (Capreolus capreolus) at sites with wildlife warning reflectors
Source: PLoS One. 2017 Sep 27;12(9):e0184761. doi: 10.1371/journal.pone.0184761 (PMC5617160; doi:10.1371/journal.pone.0184761)
Supplement: S2 Fig — Boxplots depict variation in the amount of recorded road crossings per week for each study animal. Numbers in the top row denote the amount of weeks that each animal provided data (N). Black bars indicate the median of road crossing frequency for each animal and boxes delimit the interquartile range. Colours are arbitrary. (DOCX) [file pone.0184761.s003.docx]

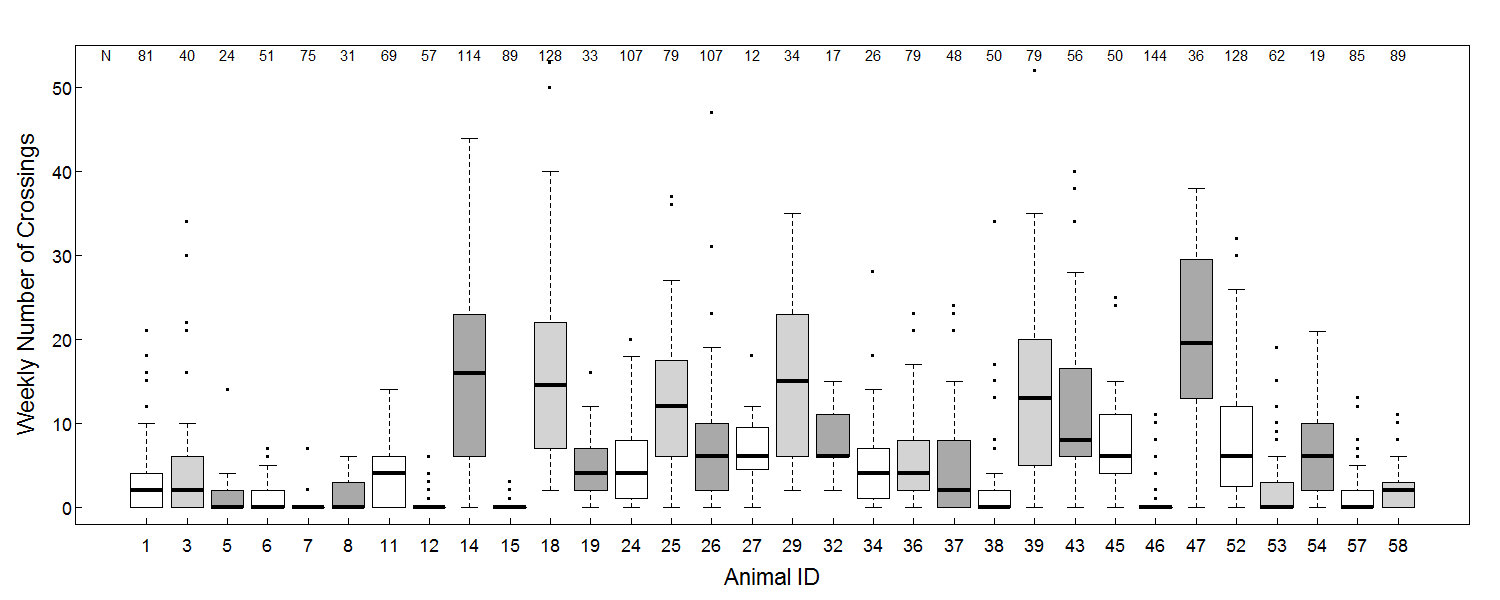


**S2 Fig. Overview of inter-individual variability in weekly crossing frequencies.** Boxplots depict variation in the amount of recorded road crossings per week for each study animal. Numbers in the top row denote the amount of weeks that each animal provided data (N). Black bars indicate the median of road crossing frequency for each animal and boxes delimit the interquartile range. Colours are arbitrary.
