# Supplementary material for: Temporal patterns in road crossing behaviour in roe deer (Capreolus capreolus) at sites with wildlife warning reflectors
Source: PLoS One. 2017 Sep 27;12(9):e0184761. doi: 10.1371/journal.pone.0184761 (PMC5617160; doi:10.1371/journal.pone.0184761)
Supplement: S3 Fig — Collision records were available for three out of five road sections used in the study. (DOCX) [file pone.0184761.s004.docx]

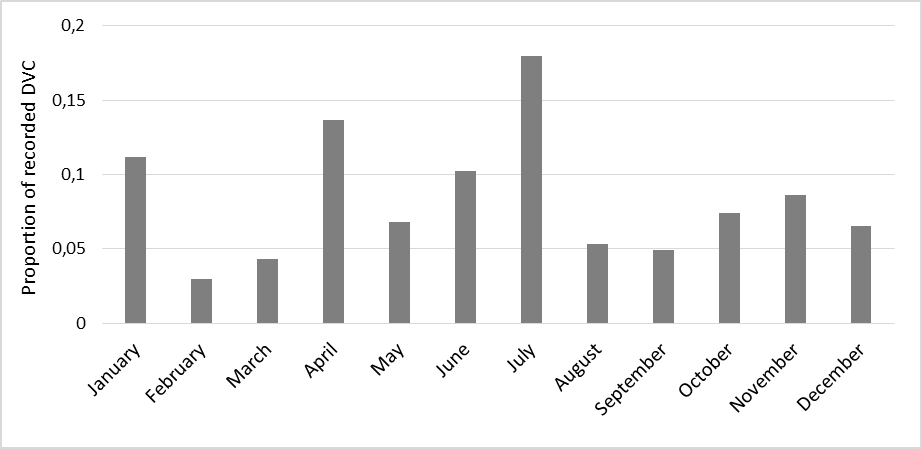


**S3 Fig. Pooled roe deer-vehicle collision records by month over the duration of the study.** Collision records were available for three out of five road sections used in the study.
