# Supplementary material for: Temporal patterns in road crossing behaviour in roe deer (Capreolus capreolus) at sites with wildlife warning reflectors
Source: PLoS One. 2017 Sep 27;12(9):e0184761. doi: 10.1371/journal.pone.0184761 (PMC5617160; doi:10.1371/journal.pone.0184761)
Supplement: S4 Fig — In addition, another 14 studied animals were found dead, but the cause of death was unclear. (DOCX) [file pone.0184761.s005.docx]

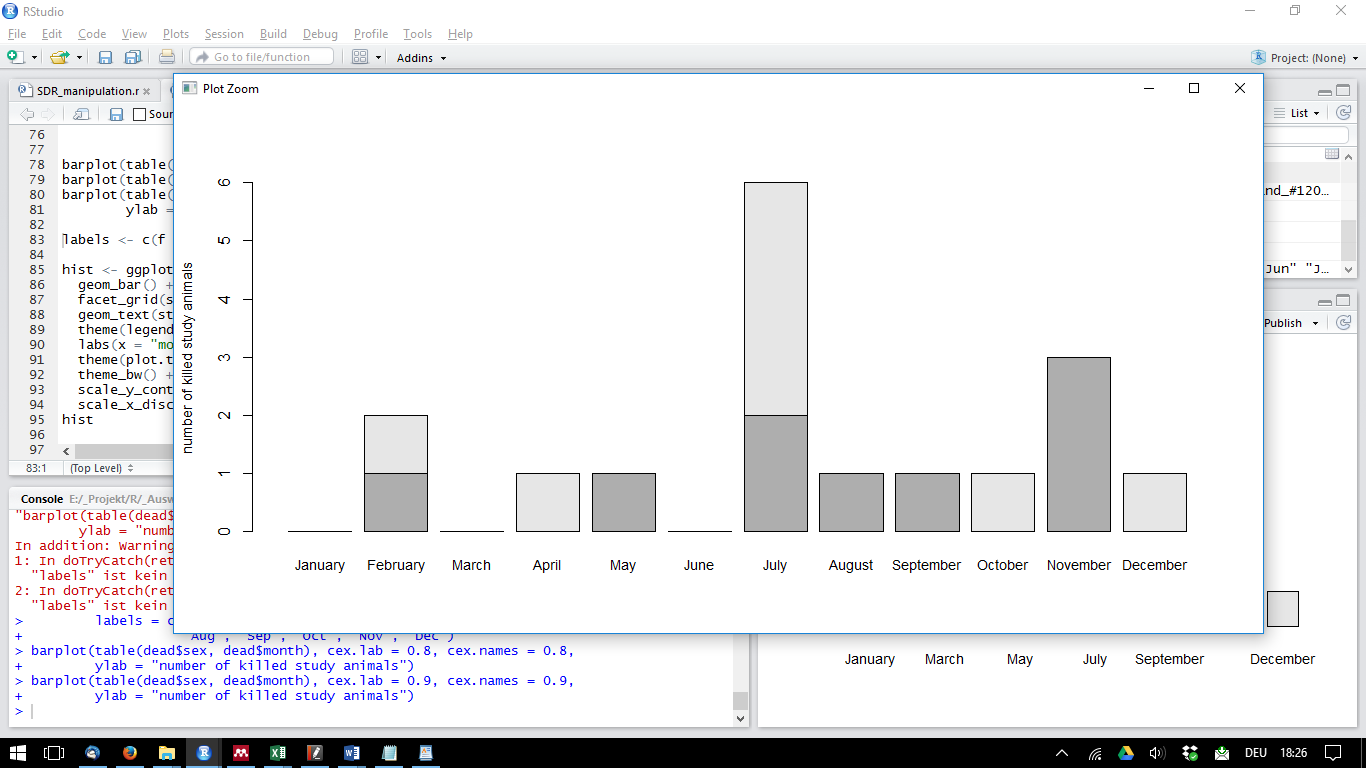


**S4 Fig. Overview of confirmed roe deer-vehicle collision involving GPS-collared studied animals (N = 17 of 46); males light grey, females grey.** In addition, another 14 studied animals were found dead, but the cause of death was unclear.
